# ZG16 promotes T-cell mediated immunity through direct binding to PD-L1 in colon cancer

**DOI:** 10.1186/s40364-022-00396-y

**Published:** 2022-07-13

**Authors:** Hui Meng, Wu Yao, Yuhui Yin, Yizhen Li, Yi Ding, Liang Wang, Mingzhi Zhang

**Affiliations:** 1 Department of Pathology, First Affiliated Hospital of Zhengzhou University, Zhengzhou, Henan China; 2 College of Public Health, Zhengzhou University, Zhengzhou, Henan China; 3 Department of Tumor Biology, H. Lee Moffitt Cancer Center and Research Institute, Tampa, FL USA; 4 Department of Oncology, First Affiliated Hospital of Zhengzhou University, Zhengzhou, Henan China

**Keywords:** ZG16, PD-L1, PD1, Colorectal cancer, T cells

## Abstract

Immunotherapy using programmed cell death 1 (PD1) inhibitors has shown great efficacy in colorectal cancer patients harboring mismatch-repair-deficient (dMMR) and microsatellite instability-high (MSI-H) alterations. We previously showed a negative correlation of zymogen granule protein 16 (*ZG16*) with programmed death-ligand 1 (*PD-L1*) expression in patients with colorectal cancer. However, how *ZG16* regulates *PD-L1* expression is unclear. In this study, we showed that *ZG16* can directly bind to glycosylated *PD-L1* through its lectin domain, leading to PD-L1 degradation. Mutations on the lectin domain of *ZG16* largely inhibit the interaction between *ZG16* and *PD-L1*. Importantly, *ZG16* overexpression suppressed tumor growth in two syngeneic mouse models through blockage of *PD-L1* expression in cancer cells meanwhile suppression of PD1 expression in T cells. We also showed that *ZG16* could improve the effect of chemotherapy and may be delivered as a protein to serve as an immune checkpoint inhibitor to promote T-cell mediated immunity.

**Supplementary Information:**

The online version contains supplementary material available at 10.1186/s40364-022-00396-y.

To the Editor,

Colorectal cancer (CRC) is a heterogeneous disease with a wide variety of genetic alterations, including mismatch-repair-deficient (dMMR) and microsatellite instability-high (MSI-H). Defects in MMR can lead to MSI-H, which can be found in many types of cancer. MSI-H or mismatch repair deficient (dMMR) tumors have an accumulation of errors in genetic sequences that are normally repeated (called microsatellites). Immunotherapy using checkpoint inhibitors that targets the PD-1/PD-L1 pathway, pembrolizumab, and nivolumab, has shown great efficacy in CRC patients harboring dMMR and MSI-H alterations, possibly attributable to a high level of immune checkpoint genes, including *PD-1, PD-L1, CTLA-4, LAG-3*, and *IDO* in such MSI-H tumors [1, 2]. Human *zymogen granule protein 16* (*ZG16*) is highly expressed in mucus-secreting cells and characterized by a Jacalin-like lectin domain [3]. We previously showed a negative correlation of *ZG16* with *PD-L1* expression in patients with CRC and blockage of *PD-L1* expression by *ZG16* in CRC cells [4]. Surprisingly, we did not observe any change on the *PD-L1* RNA after overexpression of *ZG16*, suggesting that *PD-L1* is not regulated at transcriptional level [4]. So far, how *ZG16* regulates *PD-L1* expression is unclear.

Studies have shown that the activity of *PD-L1* is regulated by N-glycosylation, and targeting glycosylated *PD-L1* (*gPD-L1*) by monoclonal antibody blocks PD-L1/PD-1 interaction resulting in *PD-L1* degradation [5, 6]. Interestingly, *ZG16* contains a lectin domain and lectins are carbohydrate-binding proteins that can specifically select for glycosylated proteins (Fig. 1a and Supplementary Fig. 1a) [7]. Based on these findings, we hypothesized that ZG16 may directly bind to glycosylated *PD-L1* through its lectin domain, leading to *PD-L1* degradation.

**Fig. 1:**
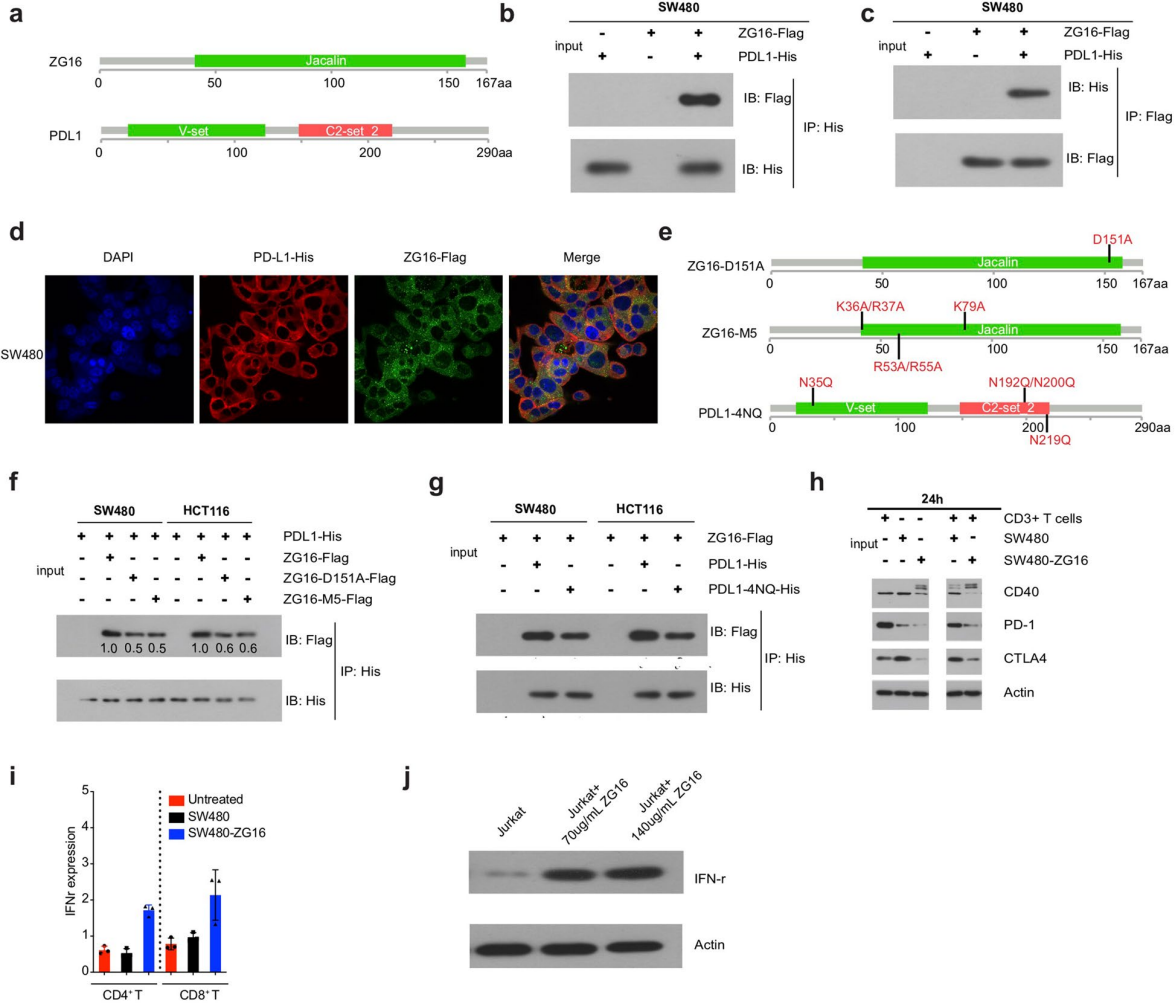
ZG16 binds to glycosylated PD-L1 through its lectin domain **a** Structure of ZG16 and PD-L1. **b-c** Co-immunoprecipitation of Flag-tagged ZG16 (ZG16-Flag) with His-tagged PD-L1 (PD-L1-His). Plasmid construct of ZG16-Flag was co-transfected with PD-L1-His into SW480 cells. Single vectors expressing each tag (Flag, His) were used as negative controls. **d** Immunofluorescence of SW480 cells transfected with ZG16-Flag and PD-L1-His, alone or in combination, for 24 h. Cells were subsequently stained with antibodies against His-tag (Red) and Flag-tag (Green) and DAPI (blue; nuclei). **e** Structure of ZG16-D151A, ZG16-M5, and PD-L1-4NQ **f** Co-immunoprecipitation of Flag-tagged ZG16 (ZG16-Flag, ZG16-D151A-Flag or ZG16-M5-Flag) with His-tagged PD-L1 (PD-L1-His). Plasmid constructs of ZG16-Flag, ZG16-D151A-Flag, or ZG16-M5-Flag were co-transfected with PD-L1-His into SW480 cells. Single vectors expressing each tag (Flag, His) were used as negative controls. **g** Co-immunoprecipitation of His-tagged PD-L1 (PDL1-His or PDL1-4NQ-His) with Flag-tagged ZG16 (ZG16-Flag). Plasmid constructs of PDL1-His or PDL1-4NQ-His were co-transfected with ZG16-Flag into SW480 or HCT118 cells. Single vectors expressing each tag (Flag, His) were used as negative controls. **h** Immunoblots of CD3^+^T cells cocultured withSW480 or SW480-ZG16 for 24 h. **i** INF-r expression of CD4^+^T cells or CD8^+^T cells cocultured withSW480 or SW480-ZG16. **j** Jurkat cells were treated with purified ZG16 protein for 48 h and then western blot was performed to detect IFNr expression

To test this hypothesis, we first build a 3D model using iCn3D to detect their interactions. The 3D modeling supported the interaction of *ZG16* with *PD-L1* (Supplementary Fig. 1b). Encouraged by these observations, we constructed two overexpression plasmids expressing Flag-tagged ZG16 and His-tagged PD-L1. To detect their direct binding in cells, we co-transfected Flag-tagged ZG16 plasmid and His-tagged PD-L1 plasmid into SW480 cells. qRT-PCR and western blot confirmed the overexpression of *ZG16* and *PD-L1* in SW480 cells after co-transfection (Supplementary Fig. 2a and b). We then performed a co-immunoprecipitation (co-IP) assay to detect the binding. Very surprisingly, we observed a direct binding between *ZG16* and *PD-L1* in SW480 cells co-transfected with two tagged plasmids (Fig. 1b and c). To exclude the possibility that this interaction between *ZG16* and *PD-L1* is cell line-specific, we performed co-IP assay in another colon cancer cell line HCT116 after co-transfection. Consistently, the direct interaction between ZG16 and *PD-L1* was observed in HCT116 (Supplementary Fig. 3a and b), indicating that *ZG16* binding to *PD-L1* is not cell line-specific. To further confirm the direct interaction between *ZG16* and *PD-L1* in colon cells, we performed a colocalization assay in two different cell lines co-transfected with two plasmids expressing Flag-tagged *ZG16* and His-tagged *PD-L1*. In consistency with the co-IP assay, we observed a direct binding between *ZG16* and *PD-L1* (Fig. 1d and Supplementary Fig. 3c). Together, these data suggest that *ZG16* can directly bind to *PD-L1* in colon cancer cells.

To determine whether the interaction between *ZG16* and *PD-L1* is dependent on the lectin domain, we constructed two Flag-tagged *ZG16* overexpression plasmids which contain mutations in the lection domain. The first plasmid contains D151A mutation (*ZG16*-D151A) and the second plasmid contains 5 different mutations (*ZG16*-M5: K36A, R37A, K79A, R53A, and R55A). We then co-transfected the two plasmids expressing different mutant *ZG16* with the plasmid expressing His-tagged *PD-L1* into SW480 and HCT116 cells and performed co-IP (Fig. 1e). Very importantly, we observed a significant reduction of the binding between *ZG16* and *PD-L1* when the mutations were introduced into the lectin domain of *ZG16*, indicating that the lectin domain is required for the interaction (Fig. 1f and Supplementary Fig. 4a-b). The fact that the interaction was not completely blocked by the mutation suggests that more mutations may be required to completely eliminate the binding. In 2016, Chia-Wei Li and his colleagues demonstrated that PD-L1 glycosylation was completely ablated in the PD-L1 4NQ mutant^5^. To determine whether the binding is dependent on the glycosylation of *PD-L1*, we introduced 4 different mutations in *PD-L1* (PD-L1-4NQ: N35Q, N192Q, N200Q, and N219Q) which has shown to affect the glycosylation of PD-L1 (Fig. 1e). Again, we observed the reduction of the binding between *ZG16* and *PD-L1* in the two cell lines with *PD-L1* mutations, suggesting that glycosylation of *PD-L1* is necessary for the binding (Fig. 1g and Supplementary Fig. 4c). Clearly, these data demonstrate that *ZG16* can directly bind to glycosylated *PD-L1* through its lectin domain.

As a secret protein primarily in the mucosa layer of the colon, we were also wondering if ZG16 is involved in the defense system, with a focus on the regulation of primary T cells. We investigated whether overexpression of *ZG16* in colon cancer cells affects the gene expression of stimulatory and inhibitory checkpoint molecules, including *CD40, PD1*, and *CTLA4*. We co-cultured primary CD3^+^ T cells with ZG16 over-expressed SW480 cells (SW480-ZG16) for different time points. Western blots showed that additional isoforms of *CD40* were induced, probably due to the ligand-binding or post-translational modification. Importantly, the expression of *PD1* and *CTLA4* was significantly decreased (Fig. 1h and Supplementary Fig. 5a). We observed an increased level of *IFN-r* in both CD4^+^ cells and CD8^+^T cells when co-cultured with the SW480-ZG16 cells (Fig. 1i and Supplementary Fig. 5b), suggesting that the T cells were activated. To confirm our finding, we treated Jurkat cells with purified ZG16 protein for 48 h and then performed FACS analysis to detect PD1 and CTLA4. Importantly, both expression of PD1 and CTLA4 were significantly decreased after ZG16 treatment in Jurkat cells (Figure S5c and S5d). in addition, we also performed western blot to detect IFNr expression in Jurkat cells. Consistently, IFNr expression were significantly increased after ZG16 treatment in Jurkat cells, indicating that the Jurkat cells were activated by ZG16 (Fig. 1j). These results support that *ZG16* serves as an immune checkpoint inhibitor to activate the T cells by blocking the gene expression of *PD1* and *CTLA4*.

Intrigued by our promising cell lines-based results, we further investigated in vivo efficacy of *ZG16* overexpression in CRC xenografts. We subcutaneously implanted murine CT26 cells and CT26 cells with *ZG16* overexpression (CT26-ZG16) into the right flank of female BALB/c mice to generate syngeneic mouse models. We followed these xenografts for 35 days. We found that *ZG16* overexpression significantly suppressed tumor growth (Fig. 2a and b). Immunohistochemistry (IHC) analysis of residual tumors showed that the *ZG16* overexpression resulted in more pronounced CD3 and significantly decreased *PD-L1* and *PD1* (Fig. 2c), suggesting that *ZG16* overexpression blocked *PD-L1* expression in cancer cells meanwhile stimulated T cell activation by suppression of *PD1* expression, which in turn contributes to the inhibition of tumor growth.

**Fig. 2:**
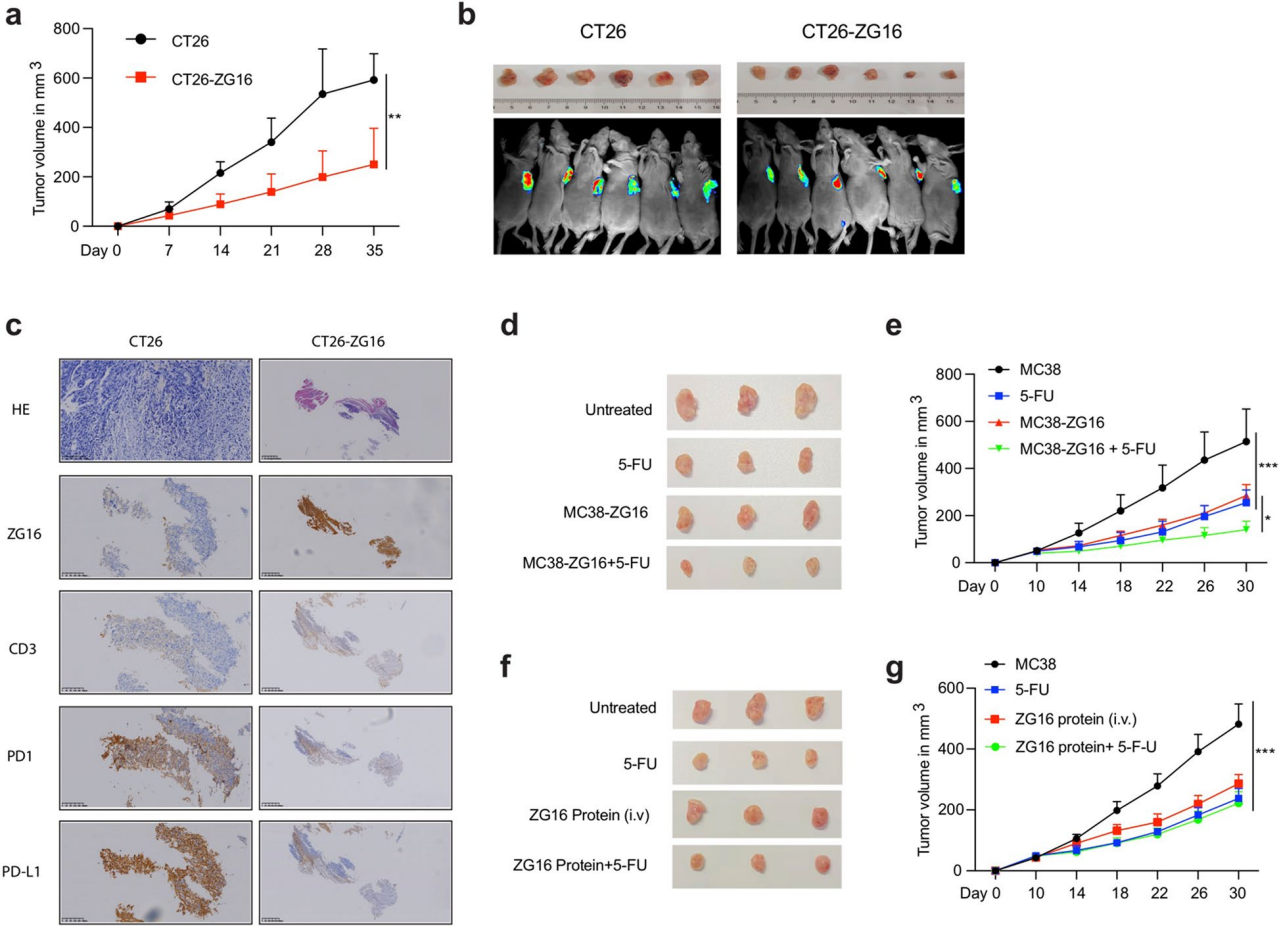
ZG16 improves the effect of chemotherapy and promotes T-cell mediated immunity **a** Growth curve of CT26 and CT26-ZG16 xenografts (*n* = 6). Data are shown as mean ± s.d.. **P < 0.01 by two-way ANOVA with Tukey’s multiple comparisons test. **b** the Tumor volume of CT26 and CT26-ZG16 xenografts. **c** H& E and IHC analysis (ZG16, CD3, PD1, PD-L1) of CT26 and CT26-ZG16 xenografts. Scale bar = 400 μm. **d-e** Tumor volume and growth curve of MC38 and MC38-ZG16 xenografts treated with PBS or 5-FU(*n* = 3). Data are shown as mean ± s.d..*P < 0.05, ***P < 0.001 by two-way ANOVA with Tukey's multiple comparisons tests. **f-g** Tumor volume and growth curve of MC38 xenografts treated with 5-FU, ZG16 protein or their combination (*n *= 3). Data are shown as mean ± s.d.***P < 0.001 by two-way ANOVA with Tukey’s multiple comparisons test

Finally, we investigated whether *ZG16* overexpression could improve the effectiveness of chemotherapy in CRC cancer xenografts. We subcutaneously implanted murine MC38 cells and MC38 cells with *ZG16* overexpression (MC38-ZG16) into the right flank of female C57BL/6 mice. The MC38-ZG16 or MC38 xenografts were treated with PBS or 5-FU for indicated time points and the mice were monitored for 30 days. Notably, *ZG16* overexpression significantly suppressed tumor growth and showed a similar effect as chemotherapy (Fig. 2d and Supplementary Fig. 6a, 7a). Most importantly, the combination of *ZG16* overexpression and 5-FU resulted in significantly greater suppression of tumor growth (Fig. 2e).

As a potential immune checkpoint inhibitor, we investigated whether ZG16 can be functional when delivered as a protein. We purified ZG16 protein and then tested the in vivo efficacy of ZG16 protein delivered by tail vein injection in MC38 xenografts (Fig. 2f and Supplementary Fig. 6b). We found that ZG16 when delivered as protein significantly inhibited tumor growth (Fig. 2g and Supplementary Fig. 6b). We did not observe a synergistic effect between single agent and their combination, probably due to the low dose of ZG16 protein (Fig. 2g). To investigate whether the T cells were involved in the tumor suppression, we measured the percentage of both CD4^+^ T cells and CD8^+^ T cells in the tumor, spleen, and blood. We observed an increased number of CD4^+^ T cells and CD8^+^ T cells in the combination group (Supplementary Fig. 7b and c). Collectively, these results demonstrate that *ZG16* could improve the effect of chemotherapy and may be delivered as a protein to serve as an immune checkpoint inhibitor to activate the T cells.

In conclusion, our study for the first time demonstrated that *ZG16* can promote T-cell mediated immunity through direct binding to glycosylated *PD-L1* in Colon Cancer. Overexpression of *ZG16* significantly suppressed tumor growth and improved the effect of chemotherapy. Most importantly from a clinical standpoint, *ZG16* can be delivered as a protein to activate the T cells by blocking the gene expression of *PD1* and *CTLA4*. We envision that our findings may also be applied to other types of cancer. Our results may lead to the discovery of novel immune checkpoint inhibitors, which will provide new routes of immunotherapy for cancer treatment.

## Supplementary Information

**Additional file 1.**

## Data Availability

The datasets used and/or analyzed during the current study are available from the corresponding author on reasonable request.

## Abbreviations

ZG16: Zymogen granule protein 16
CRC: Colorectal cancer
PD-L1: Programmed cell death-ligand 1
PD-L1: Programmed death-ligand 1
