# Supplementary material for: ZG16 promotes T-cell mediated immunity through direct binding to PD-L1 in colon cancer
Source: Biomark Res. 2022 Jul 13;10:47. doi: 10.1186/s40364-022-00396-y (PMC9281127; doi:10.1186/s40364-022-00396-y)
Supplement: Supplementary file 1 — Additional file 1. [file 40364_2022_396_MOESM1_ESM.pdf]

Supplementary Information

ZG16 promotes T-cell mediated immunity through direct binding to PD-L1 in Colon Cancer

Hui Meng[1*], Wu Yao[2], Yuhui Yin[1], Yizhen Li[1], Yi Ding[1], Liang Wang[3], Mingzhi Zhang[4*]

Author details

[1]Department of Pathology, First Affiliated Hospital of Zhengzhou University, Zhengzhou, Henan, China. [2]College of Public Health, Zhengzhou University, Zhengzhou, Henan, China. [3]Department of Tumor Biology, H. Lee Moffitt Cancer Center and Research Institute, Tampa, Florida, USA. [4]Department of Oncology, First Affiliated Hospital of Zhengzhou University, Zhengzhou, Henan, China.

*These two authors contributed equally to this work

Material and Methods

Cell culture

CRC cell lines (SW480, HCT116, CT26, and MC38) were obtained from American Type Culture Collection (ATCC, Manassas, VA). All cells were cultured in RPMI-1640 media supplemented with 10% fetal bovine serum (FBS) and 1% penicillin/streptomycin. Cells were maintained in a humidified incubator adjusted with 5% CO2 at 37 °C. All cells were authenticated by DNA fingerprinting using highly-polymorphic short tandem repeat (STR) analysis and confirmed free from mycoplasma contamination.

Vectors and lentivirus production

Overexpression vectors for ZG16-FLAG, ZG16D151A-FLAG, ZG16-M5, PDL1-His, or PDL1-4NQ-His were constructed by Gibson assembly (NEB) (**Supplementary sequence 1**). Lentiviral vectors for ZG16orPD-L1 overexpression were constructed by Gibson assembly (NEB). The lentivirus was produced as described.[8] Briefly, H293T cells were plated in a 10 cm dish at 30% confluence in DMEM plus 10% FBS. For each dish, 9 µg of lentiviral vector, 0.9 µg of pVSVg (Addgene, Cat. #8584), 9 µg of psPAX2 (Addgene, Cat. #12260) were mixed and transfected to the H293T cells. Lentivirus was collected 48 hours later by filtering through a 0.45 µm strainer. Both CT26 and MC38 cells were transfected with 1×10^6 IFU/ml lentivirus 24 h. Transduced cells were selected using 2 µg/ml puromycin (Sigma-Aldrich) for 7 days.

Co-immunoprecipitation assay

Plasmid construct of either ZG16-FLAG, ZG16D151A-FLAG, or ZG16-M5 was co-transfected with PDL1-His or PDL1-4NQ-His into SW480 or HCT116 cells. Empty vectors were used as negative controls. Cells were harvested and lysed in RIPA buffer after 48 hours transfection with 1:50 Roche Complete protease inhibitor for 30 min on ice and cleared at 12,000 g for 15 min at 4°C. The cell lysate was incubated with anti-FLAG (Abclonal, AE005 or AE063, 1:200) or anti-His (Abclonal, AE068 or AE003,1:200) at 4°C overnight, then incubated with 150 µl Protein A/G-coated magnetic beads for 2 hours at 4°C. 50 µl 1 x loading buffer was added to the beads to extract proteins for SDS-PAGE.

Western Blot Analysis

Equal amounts of protein lysates (5 µg/lane) were run on 4%-20% gradient SDS-polyacrylamide gels (Bio-Rad Laboratories, Inc., Hercules, CA, USA) and then were transferred to Immobilon-P

nitrocellulose membranes (Millipore, Billerica, MA, USA). The membranes were probed with rabbit anti-PD1 antibody (Wanlbio, WL04252, 1:1000), or anti-CD40 antibody (Wanlbio, WL0154, 1:500), or anti-CTLA4 antibody (Wanlbio, WL03020, 1:500), and mouse anti-β-actin (Wanlbio, WL01372, 1:500) in 4°C overnight after being blocked with 5% non-fat milk. The membranes were then incubated with species-specific conjugated secondary antibodies (GE Dharmacon) at room temperature for 1 hour. An ECL blotting analysis system (Amersham Pharmacia Biotech, Piscataway, NJ, USA) was used for detecting protein expression.

T Cell Co-Culture and INF-r detection by FlowCytometry

Fresh blood was collected from 4 health donors and $CD3^{+}$T, $CD4^{+}$T, and $CD8^{+}$T cells were isolated. SW480 cells were grown at 37° in 5% CO2 humidified air in RPMI-1640 medium that contained 10% fetal bovine serum, 100 U/ml penicillin, and 100 μg/ml streptomycin. In co-cultures, the cells were grown in a mixed medium (1: 1) of SW480 and NK cells. SW480-ZG16 or SW480 cells were first seeded at 5×10^{3} cells per plate, and then cultured for indicated time points. Then, the T cells (5×10^{3} cells/well) were added to the plate. The cells were pelleted by centrifugation and INF-r expression was determined by flow cytometry.

Real-Time Quantitative PCR

Total RNA was extracted from Paraffined tissues using the RNeasy FFPE Kit (QIAGEN) and cDNA was synthesized using the SuperScript VILO Master Mix (ThermoFisher Scientific) following the manufacturer’s instructions. 5μl cDNA was used in the PCR with ZG16 or GAPDH primers mixed with Power SYBR Green PCR mix at a final volume of 20 μl in triplicate (Applied Biosystems). qPCR analysis was done using the ABI StepOne plus Software Real-Time PCR system. Gene expression of target genes was normalized against ACTB and compared among different groups by the ΔΔCT method.

Immunofluorescence and immunohistochemistry

SW480 and HCT116 cells are transfected in six-well format via Lipofectamine 2000 (Invitrogen) using the manufacturer's suggested protocol with ZG16-FLAG, or PD-L1-HIS or their combination. 72 h following transfection, cells were washed with 1× PBS and fixed with 4% formaldehyde in 1× PBS for 15 min at room temperature. Following blocking (blocking solution: 2% BSA, 0.3% Triton X-100, within 1× PBS), samples were stained with anti-FLAG or anti-HIS antibodies. VECTASHIELD mounting medium with DAPI (Vector Laboratories, H-1200) was used to stain the

nuclei and to mount the samples on the slide. Images were taken with ZEISS LSM 710 Confocal Microscope System.

For immunohistochemistry, paraffined tissues were sectioned at a thickness of 4 μm and subsequently stained with hematoxylin and eosin (H&E) and PD-L1 antibody (Spring Bio-science, SP142[9, 10][9, 10]), or PD1 (Wanlbio, WL04252) or ZG16 (Proteintech, 17397-1-AP).

In vivo mouse study

All animal experiments were conducted with the approval of the Institutional Animal Care and Use Committee (First Affiliated Hospital of Zhengzhou University). For xenografts in BALB/C mice, suspensions of 3 million CT26-ZG16 or CT26 cells (in PBS) were mixed 1:1 with BD Matrigel Basement Membrane Matrix (Cat. #356231; Corning, NY, USA) were subcutaneously inoculated in the right flank of BALB/C mice. For xenografts in C57BL/6 mice, suspensions of 3 million MC38-ZG16 or MC38 cells (in PBS) were mixed 1:1 with BD Matrigel Basement Membrane Matrix (Cat. #356231; Corning, NY, USA) were subcutaneously inoculated in the right flank of C57BL/6 mice.

Animals were assessed for 4-6 weeks after the inoculation for tumor incidence and growth and then were sacrificed. Tumor volume was measured using the formula: Tumor volume = $1/2(\text{length} \times \text{width}^2)$.

Statistical Analysis

Statistical analyses were performed using GraphPad Prism 7.01 (GraphPad Software Inc.) unless otherwise indicated. Group allocation was performed randomly. In all studies, data represent biological replicates (n) and are depicted as mean ± s.d. or mean ± SEM as indicated in the figure legends. Comparison of mean values was conducted with unpaired, two-tailed Student’s *t*-test, one-way ANOVA, or two-way ANOVA with Tukey's multiple comparisons test as indicated in the figure legends. In all analyses, *P* values less than 0.05 were considered statistically significant.

Supplementary Fig. 1. The binding model between ZG16 and PD-L1.

a, Lectin domain in ZG16 protein.

b, Binding model between ZG16 and PD-L1 was generated by iCn3D. The red color indicates PD-L1 protein and the blue color indicated ZG16 protein.

Supplementary Fig. 2. Overexpression of ZG16 and PD-L1 in SW480 cells.

a, ZG16 and PD-L1 expression determined by qRT-PCR in SW480 cells transfected with ZG16-Flag plasmid or PD-L1-his plasmid.

b, Immunoblots of ZG16 and PD-L1 expression by western blot in SW480 cells transfected with ZG16-Flag plasmid or PD-L1-his plasmid.

Supplementary Fig. 3. ZG16 binds to PD-L1.

a-b, Co-immunoprecipitation of Flag-tagged ZG16 (ZG16-Flag) with His-tagged PD-L1 (PD-L1-His). Plasmid construct of ZG16-Flag was co-transfected with PD-L1-His into HCT116 cells. Single vectors expressing each tag (Flag, His) were used as negative controls.

c, Immunofluorescence of HCT116 cells transfected with ZG16-Flag and PD-L1-His, alone or in combination, for 24 h. Cells were subsequently stained with antibodies against His-tag (Red) and Flag-tag (Green) and DAPI (blue; nuclei).

Supplementary Fig. 4. ZG16 binds to glycosylated PD-L1 through its lectin domain.

a, Structure of ZG16-D151A, ZG16-M5, and PD-L1-4NQ.

b, Co-immunoprecipitation of Flag-tagged ZG16 (ZG16-Flag, ZG16-D151A-Flag or ZG16-M5-Flag) with His-tagged PD-L1 (PD-L1-His). Plasmid constructs of ZG16-Flag, ZG16-D151A-Flag, or ZG16-M5-Flag were co-transfected with PD-L1-His into SW480 cells. Single vectors expressing each tag (Flag, His) were used as negative controls.

c, Co-immunoprecipitation of His-tagged PD-L1 (PDL1-His or PDL1-4NQ-His) with Flag-tagged ZG16 (ZG16-Flag). Plasmid constructs of PDL1-His or PDL1-4NQ-His were co-transfected with

ZG16-Flag into SW480 or HCT118 cells. Single vectors expressing each tag (Flag, His) were used as negative controls.

Supplementary Fig. 5. ZG16 overexpression activates T cells.

a, Immunoblots of $CD3^{+}$T cells cocultured withSW480 or SW480-ZG16 for 48hours.

b, The percentage of proliferating $CD3^{+}$ T cells cocultured with SW480 or SW480-ZG16 was quantified by FACS. The "-" line denotes CD3+ T cells only.

c-d,Jurkat cells were treated with purified ZG16 protein for 48hours and then FACS analysis was performed to detect the expression of PD1 (c) and CTLA4 (d).

Supplementary Fig. 6. In vivo efficacy of ZG16.

a, Tumor volume of MC38 and MC38-ZG16 xenografts treated with PBS or 5-FU(n=3).

b, the Tumor volume of MC38 xenografts treated with 5-FU, ZG16 protein, or their combination (n=3).

Supplementary Fig. 7. ZG16 overexpression improves the effect of chemotherapy and may serve as an immune checkpoint inhibitor.

a, Immunoblots of MC38 and MC38-ZG16 xenografts treated with PBS or 5-FU(n=3).

b-c, The percentage of $CD4^{+}$ T cells and $CD8^{+}$ T cells in MC38 and MC38-ZG16 xenografts treated with PBS, 5-FU, or their combinations.

Supplementary Sequences 1. Sequences of plasmid used in this study

ZG16 :

ATGTTGACAGTCGCTCTCCTAGCCCTTCTCTGTGCCTCAGCCTCTGGCAATGCCATTCAGGCCAGGTCTTCCTCCTAT
AGTGGAGAGTATGGAAGTGGTGGTGGAAAGCGATTCTCTCATTCTGGCAACCAGTTGGACGGCCCCATCACCGCC
CTCCGGGTCCGAGTCAACACATACTACATCGTAGGTCTTCAGGTGCGCTATGGCAAGGTGTGGAGCGACTATGTG
GGTGGTCGCAACGGAGACCTGGAGGAGATCTTTCTGCACCCTGGGGAATCAGTGATCCAGGTTTCTGGGAAGTAC
AAGTGGTACCTGAAGAAGCTGGTATTTGTGACAGACAAGGGCCGCTATCTGTCTTTTGGGAAAGACAGTGGCACA
AGTTTCAATGCCGTCCCCTTGCACCCCAACACCGTGCTCCGCTTCATCAGTGGCCGGTCTGGTTCTCTCATCGATGC
CATTGGCCTGCACTGGGATGTTTACCCCACTAGCTGCAGCAGATGCTGA

ZG16-D151A :

ATGTTGACAGTCGCTCTCCTAGCCCTTCTCTGTGCCTCAGCCTCTGGCAATGCCATTCAGGCCAGGTCTTCCTCCTAT
AGTGGAGAGTATGGAAGTGGTGGTGGAAAGCGATTCTCTCATTCTGGCAACCAGTTGGACGGCCCCATCACCGCC
CTCCGGGTCCGAGTCAACACATACTACATCGTAGGTCTTCAGGTGCGCTATGGCAAGGTGTGGAGCGACTATGTG
GGTGGTCGCAACGGAGACCTGGAGGAGATCTTTCTGCACCCTGGGGAATCAGTGATCCAGGTTTCTGGGAAGTAC
AAGTGGTACCTGAAGAAGCTGGTATTTGTGACAGACAAGGGCCGCTATCTGTCTTTTGGGAAAGACAGTGGCACA
AGTTTCAATGCCGTCCCCTTGCACCCCAACACCGTGCTCCGCTTCATCAGTGGCCGGTCTGGTTCTCTCATCGCGGC
CATTGGCCTGCACTGGGATGTTTACCCCACTAGCTGCAGCAGATGCTGA

ZG16-M5 :

ATGTTGACAGTCGCTCTCCTAGCCCTTCTCTGTGCCTCAGCCTCTGGCAATGCCATTCAGGCCAGGTCTTCCTCCTAT
AGTGGAGAGTATGGAAGTGGTGGTGGAGCGGCGTTCTCTCATTCTGGCAACCAGTTGGACGGCCCCATCACCGCC
CTCGCGGTCGCGGTCAACACATACTACATCGTAGGTCTTCAGGTGCGCTATGGCAAGGTGTGGAGCGACTATGTG
GGTGGTGCGAACGGAGACCTGGAGGAGATCTTTCTGCACCCTGGGGAATCAGTGATCCAGGTTTCTGGGAAGTA
CAAGTGGTACCTGAAGAAGCTGGTATTTGTGACAGACAAGGGCCGCTATCTGTCTTTTGGGAAAGACAGTGGCAC
AAGTTTCAATGCCGTCCCCTTGCACCCCAACACCGTGCTCCGCTTCATCAGTGGCCGGTCTGGTTCTCTCATCGATG
CCATTGGCCTGCACTGGGATGTTTACCCCACTAGCTGCAGCAGATGCTGA

PD-L1 :

ATGAGGATATTTGCTGTCTTTATATTCATGACCTACTGGCATTTGCTGAACGCATTTACTGTCACGGTTCCCAAGGA
CCTATATGTGGTAGAGTATGGTAGCAATATGACAATTGAATGCAAATTCCCAGTAGAAAAACAATTAGACCTGGCT
GCACTAATTGTCTATTGGGAAATGGAGGATAAGAACATTATTCAATTTGTGCATGGAGAGGAAGACCTGAAGGTT
CAGCATAGTAGCTACAGACAGAGGGCCCGGCTGTTGAAGGACCAGCTCTCCCTGGGAAATGCTGCACTTCAGATC
ACAGATGTGAAATTGCAGGATGCAGGGGTGTACCGCTGCATGATCAGCTATGGTGGTGCCGACTACAAGCGAATT
ACTGTGAAAGTCAATGCCCCATACAACAAAATCAACCAAAGAATTTTGGTTGTGGATCCAGTCACCTCTGAACATG
AACTGACATGTCAGGCTGAGGGCTACCCCAAGGCCGAAGTCATCTGGACAAGCAGTGACCATCAAGTCCTGAGTG
GTAAGACCACCACCACCAATTCCAAGAGAGAGGAGAAGCTTTTCAATGTGACCAGCACACTGAGAATCAACACAA

CAACTAATGAGATTTTCTACTGCACTTTTAGGAGATTAGATCCTGAGGAAAACCATACAGCTGAATTGGTCATCCC
AGAACTACCTCTGGCACATCCTCCAAATGAAAGGACTCACTTGGTAATTCTGGGAGCCATCTTATTATGCCTTGGTG
TAGCACTGACATTCATCTTCCGTTTAAGAAAAGGGAGAATGATGGATGTGAAAAAATGTGGCATCCAAGATACAA
ACTCAAAGAAGCAAAGTGATACACATTTGGAGGAGACGTAA

PD-L1-4NQ :

ATGAGGATATTTGCTGTCTTTATATTCATGACCTACTGGCATTTGCTGAACGCATTTACTGTCACGGTTCCCAAGGA
CCTATATGTGGTAGAGTATGGTAGCCAGATGACAATTGAATGCAAATTCCCAGTAGAAAAACAATTAGACCTGGCT
GCACTAATTGTCTATTGGGAAATGGAGGATAAGAACATTATTCAATTTGTGCATGGAGAGGAAGACCTGAAGGTT
CAGCATAGTAGCTACAGACAGAGGGCCCGGCTGTTGAAGGACCAGCTCTCCCTGGGAAATGCTGCACTTCAGATC
ACAGATGTGAAATTGCAGGATGCAGGGGTGTACCGCTGCATGATCAGCTATGGTGGTGCCGACTACAAGCGAATT
ACTGTGAAAGTCAATGCCCCATACAACAAAATCAACCAAAGAATTTTGGTTGTGGATCCAGTCACCTCTGAACATG
AACTGACATGTCAGGCTGAGGGCTACCCCAAGGCCGAAGTCATCTGGACAAGCAGTGACCATCAAGTCCTGAGTG
GTAAGACCACCACCACCAATTCCAAGAGAGAGGAGAAGCTTTTCCAGGTGACCAGCACACTGAGAATCCAGACAA
CAACTAATGAGATTTTCTACTGCACTTTTAGGAGATTAGATCCTGAGGAACAGCATACAGCTGAATTGGTCATCCC
AGAACTACCTCTGGCACATCCTCCAAATGAAAGGACTCACTTGGTAATTCTGGGAGCCATCTTATTATGCCTTGGTG
TAGCACTGACATTCATCTTCCGTTTAAGAAAAGGGAGAATGATGGATGTGAAAAAATGTGGCATCCAAGATACAA
ACTCAAAGAAGCAAAGTGATACACATTTGGAGGAGACGTAA
